# Combination studies with gemcitabine in the treatment of non-small-cell lung cancer.

**DOI:** 10.1038/bjc.1998.749

**Published:** 1998

**Authors:** W. P. Steward

**Affiliations:** University Department of Oncology, Leicester Royal Infirmary, UK.

## Abstract

Phase II studies have confirmed gemcitabine (GEMZAR) to be an active single agent in treating non-small-cell lung cancer (NSCLC), with response rates averaging 21%. Toxicity, including myelosuppression, is mild, making gemcitabine an attractive agent to consider in combination regimens. Most experience with gemcitabine in combination has been with cisplatin. Five phase II studies have been performed using different scheduling and dosage regimens. Response rates varied from 38% to 54% and median survival was 8.4-14.3 months. This combination was well tolerated and required minimal hospitalization. Haematological toxicity of short duration was dose limiting, with thrombocytopenia WHO grades 3/4 in 16-52% of patients and neutropenia in 36-58%. Nausea and vomiting occurred with cisplatin. Ifosfamide has been combined with gemcitabine in a phase I/II study. Based on phase I data, ifosfamide 1500 mg m(-2)day(-1) was chosen for the phase II study. The overall response rate was 32%. Toxicity was mild and was mainly related to short-lived myelosuppression. In summary, the favourable toxicity profile of single-agent gemcitabine enables its safe combination with other active agents in the treatment of NSCLC. The combination with cisplatin appears particularly encouraging, and a phase III study comparing this combination with standard chemotherapy regimens is planned. The combination of gemcitabine with radiotherapy is also under investigation.


British Journal of Cancer (1998) 78(Supplement 3), 15–19
© 1998 Cancer Research Campaign

### Combination studies with gemcitabine in the treatment of non-small-cell lung cancer

**WP Steward**

University Department of Oncology, Leicester Royal Infirmary, Leicester LE1 5WW, UK

**Summary** Phase II studies have confirmed gemcitabine (GEMZAR®) to be an active single agent in treating non-small-cell lung cancer (NSCLC), with response rates averaging 21%. Toxicity, including myelosuppression, is mild, making gemcitabine an attractive agent to consider in combination regimens. Most experience with gemcitabine in combination has been with cisplatin. Five phase II studies have been performed using different scheduling and dosage regimens. Response rates varied from 38% to 54% and median survival was 8.4–14.3 months. This combination was well tolerated and required minimal hospitalization. Haematological toxicity of short duration was dose limiting, with thrombocytopenia WHO grades 3/4 in 16–52% of patients and neutropenia in 36–58%. Nausea and vomiting occurred with cisplatin. Ifosfamide has been combined with gemcitabine in a phase I/II study. Based on phase I data, ifosfamide 1500 mg $m^{-2}$ $day^{-1}$ was chosen for the phase II study. The overall response rate was 32%. Toxicity was mild and was mainly related to short-lived myelosuppression. In summary, the favourable toxicity profile of single-agent gemcitabine enables its safe combination with other active agents in the treatment of NSCLC. The combination with cisplatin appears particularly encouraging, and a phase III study comparing this combination with standard chemotherapy regimens is planned. The combination of gemcitabine with radiotherapy is also under investigation.

**Keywords:** non-small-cell lung cancer; gemcitabine; combination therapy

Lung cancer is now the most common cause of cancer death in North America in both men and women, having recently overtaken breast cancer in women. The three main histological subtypes of non-small-cell lung cancer (NSCLC) – squamous, adenocarcinoma and large cell – account for over 80% of the 170 000 cases of bronchial carcinoma diagnosed each year in the USA. It is predominantly a disease of elderly patients, many of whom have concomitant respiratory and cardiovascular pathology.

Until recently, only four agents with a response rate of more than 15% in NSCLC were available: cisplatin, ifosfamide, vindesine and mitomycin C. The toxicities of these drugs are significant and, as the survival time for such patients has typically been short, their value has been questioned and chemotherapy has usually been avoided. In NSCLC, it is important that treatment regimens are well tolerated and require the minimum amount of hospitalization and, as a result, new agents with greater activity and fewer side-effects have been sought in recent years.

### NEW APPROACHES TO THE TREATMENT OF NSCLC

Surgery is the only curative treatment for NSCLC. Unfortunately, over 70% of patients present with advanced stages of disease and are inoperable. In these cases, chemotherapy can provide useful palliation of symptoms and modest prolongation of survival. Several promising new agents have recently become available and appear to offer advantages over established therapy in a variety of solid tumours, either in terms of greater activity or reduced toxicity.

*Correspondence to:* WP Steward

The camptothecin analogues, topotecan and irinotecan, act by inhibiting topoisomerase 1, an enzyme involved in DNA replication. Topoisomerase 1 binds to DNA during replication and causes a single-strand break in the helix, allowing relaxation of the tightly coiled configuration. Once relaxation has taken place, the nick is repaired and the enzyme dissociates from the helix. Topoisomerase 1 inhibitors prevent the resealing of the DNA strand, thus preventing DNA replication and causing cell death. Irinotecan has shown impressive response rates in clinical trials, particularly in patients with colorectal cancer (Armand et al, 1995).

Another group of agents that have shown activity in the treatment of solid tumours are the taxoids. These prevent cell division by binding to the microtubules formed during this process, forming very stable complexes and preventing mitosis. Paclitaxel has demonstrated significant single-agent activity against NSCLC in clinical trials, with response rates of 24% (Murphy et al, 1993).

Antimetabolites are agents that interfere with cell function as a result of their structural resemblance to compounds essential for cell metabolism (Clarke et al, 1991). As information on nucleotide synthesis in dividing cells has emerged, new targets for chemotherapeutic intervention have been identified, leading to the development of drugs with greater selectivity for tumour cells. Such agents can act in a variety of ways. Methotrexate, one of the earliest antimetabolites discovered, is an inhibitor of dihydrofolate reductase and leads to depletion of reduced folates within the cell (Jolivet et al, 1983). The thymidylate synthase inhibitors, another class of antimetabolite, lead to decreased thymidine biosynthesis and, ultimately, to cell death (Rustum et al, 1997).

Nucleoside analogues are also under active investigation (Perigaud et al, 1992). These are agents that resemble the nucleosides used in DNA and RNA synthesis. One example is cytosine arabinoside, an analogue of the nucleoside deoxycytidine, which is used to treat adult myelogenous leukaemia. It has no activity

**Table 1** Gemcitabine–cisplatin phase II trials: patient characteristics and schedules used

| Study | UK/France | South Africa | Italy | USA | Spain |
|---|---|---|---|---|---|
| Gemcitabine dose (mg $m^{-2}$) (days 1,8,15, every 28) | 1000 | 1000 | 1000 | 1000 | 1200 |
| Cisplatin dose (mg $m^{-2}$) (schedule) | 100 (day 15) | 100 (day 15) | 100 (day 2) | 100 (day 1) | 100 (day 15) |
| Patients entered/evaluable | 60/52 | 53/50 | 48/48 | 30/26 | 40/40 |
| Age range (median)(years) | 39–74 (59) | 35–74 (56) | 37–70 (60) | 37–74 (62) | (64) |
| Stage III/IV | 41/19 | 33/20 | 21/5 | 5/21 | 11/8 |
| Histology | | | | | N/A |
| Squamous | 30 | 26 | 22 | 10 | |
| Adenocarcinoma | 20 | 20 | 21 | 11 | |
| Large cell | 9 | 3 | 5 | 6 | |
| Unspecified | 1 | 4 | 0 | 3 | |

N/A, data not available.

against solid tumours (Keating et al, 1982). Gemcitabine is a difluorinated analogue of cytosine arabinoside, which differs from the parent compound in having increased membrane permeability and affinity for deoxycytidine kinase (Heinemann et al, 1988). It is incorporated into DNA in place of deoxycytidine, and one further nucleoside is incorporated before DNA synthesis is terminated. This mechanism, known as 'masked chain termination', appears to prevent excision repair of the altered DNA sequence by proof-reading enzymes and thus overcomes one important potential mechanism for developing resistance. In vivo, gemcitabine has shown high activity against a range of human xenografts, including breast, colon, pancreatic and lung cancers (Merriman et al, 1996).

### CLINICAL TRIALS OF GEMCITABINE IN NSCLC

Phase I and II studies have shown that gemcitabine has single-agent activity in the treatment of NSCLC, with average response rates of 20% (Kaye, 1994). The drug is well tolerated, with few serious side-effects. Those that were seen included mild leucopenia and thrombocytopenia, skin rashes and transient increases in transaminase levels. These findings make gemcitabine an excellent candidate for combination with other anti-cancer agents, with the hope of producing increased activity and acceptable toxicity.

### COMBINATION OF GEMCITABINE AND CISPLATIN

Cisplatin is currently the most frequently used agent incorporated into chemotherapy regimens. Its use is associated with a modest survival benefit compared with best supportive care. It is thought to work by forming inter- and intra-strand cross-links with DNA (Roberts and Pascoe, 1972). Unfortunately, tumour resistance develops, and an important mechanism appears to be the recognition and excision of cross-links by proof-reading enzymes. Preclinical experiments have shown that the combination of cisplatin and gemcitabine has synergistic activity against human ovarian and head and neck squamous cancer cell lines in mice, with a variable effect that depends on the schedule used (Peters et al, 1995). Clinical trials have thus been initiated to evaluate the combination in humans, and additional combinations may be worth exploring to fully exploit the experimental synergy.

**Table 2** Gemcitabine–cisplatin phase II trials: toxicities

| | Patients (%) | | | |
|---|---|---|---|---|
| Toxicity WHO grade 3/4 | Day 2 Italy | Day 15 UK/France | Day 15 South Africa | Day 15 Spain |
| Thrombocytopenia | 52 | 21 | 21 | 16 |
| Neutropenia | 36 | 58 | 58 | 56 |
| Transaminases | 0 | 4 | N/A | N/A |
| Nausea/vomiting | 27 | 64 | 64 | 28 |
| Alopecia | 6 | 8 | 8 | N/A |

N/A, data not available.

**Table 3** Gemcitabine–cisplatin phase II trials: responses and survival

| | UK/ France | South Africa | Italy | USA | Spain |
|---|---|---|---|---|---|
| Evaluable for response (patients) | 52 | 50 | 48 | 26 | 40 |
| Complete response (patients) | 0 | 2 | 1 | 1 | 0 |
| Partial response (patients) | 20 | 24 | 25 | 10 | 19 |
| Overall response rate (%) | 38 | 52 | 54 | 42 | 48 |
| Median survival (months) | 10.2 | 13 | 14.3 | 8.4 | 10.4 |
| 1-Year survival (%) | 40 | 61 | 59 | 37 | 35 |

Five phase II trials have investigated the effect of scheduling on toxicities and efficacy in NSCLC. In four of these studies, gemcitabine (1000 mg $m^{-2}$) was given on a weekly basis for 3 weeks and cisplatin (100 mg $m^{-2}$) was given once during the 28-day cycle, on different days in three trials. The schedules and patient characteristics for all five trials are summarized in Table 1.

In a sequential phase I/II study carried out in France and the UK (Steward et al, 1996), patients with inoperable, progressive NSCLC were given gemcitabine (1000 mg $m^{-2}$) on days 1, 8 and 15 of a 28-day cycle. Escalating doses of cisplatin (60–100 mg $m^{-2}$) were administered on day 15 of each cycle, immediately after the gemcitabine injection. The phase I portion of the trial identified the maximum-tolerated dose of cisplatin as 100 mg $m^{-2}$. The study was expanded into a full phase II trial including 60 patients. Of the 52 evaluable patients, partial responses were seen in 20, for an overall response rate of 38%. Neutropenia and thrombocytopenia, the most commonly observed

© Cancer Research Campaign 1998

side-effects (Table 2), were short lived and uncomplicated. Grade 3 nausea and vomiting occurred on day 15 in 50% of patients, but was not any worse than that seen with cisplatin alone. No renal or hepatotoxicities were seen in these patients and alopecia was uncommon. The median survival was 10.2 months and the 1-year survival rate was 40% (Sandler et al, 1996).

In a similar study carried out in South Africa by Abratt and colleagues (1997), 53 patients were given gemcitabine (1000 mg $m^{-2}$) on days 1, 8 and 15 of a 28-day cycle. Cisplatin (100 mg $m^{-2}$) was administered on day 15 of each cycle. The median number of cycles administered was four (range one to six). Fifty patients were evaluable for response and 53 for toxicity. The main toxicities observed were haematological, in particular neutropenia and thrombocytopenia. WHO grades 3 and 4 neutropenia were seen in 20 and 10 patients, respectively, and grades 3 and 4 thrombocytopenia in nine and four patients respectively. Other toxicities are summarized in Table 2. Complete responses were seen in two patients and partial responses in 24, for an overall response rate of 52%. The median survival duration was 13 months and the 1-year survival rate was 61%.

Two other phase II studies have been carried out that investigated the effect of giving cisplatin on different days of the schedule. The first of these involved 48 patients with stage III or IV unresectable NSCLC and was carried out in several centres in Italy (Crinò et al, 1997). Cisplatin (100 mg $m^{-2}$) was given on day 2 of each cycle. Haematological toxicities were the most frequently observed side-effects; WHO grade 3/4 thrombocytopenia was seen in 52% of patients and grade 3 or 4 neutropenia in 36%. Of 48 evaluable patients, one complete and 25 partial responses were observed, giving an overall response rate of 54%. The median response duration was 14.3 months. Ten responses lasting longer than 60 weeks were seen.

The final phase II trial using gemcitabine 1000 mg $m^{-2}$ was carried out in the USA by the Hoosier Oncology Group (Sandler et al, 1995). Cisplatin (100 mg $m^{-2}$) was given on day 1 of a 28-day cycle to 30 patients, 26 of whom were evaluable for response. Toxicities were mainly haematological, and it was frequently necessary to omit gemcitabine on days 8 and/or 15 of the cycle. One complete and ten partial responses were documented, giving an overall response rate of 42%. The median survival was 8.4 months and 37% of patients were alive at 1 year (Sandler et al, 1996).

A Spanish study investigated the effects of higher doses of gemcitabine in combination with 100 mg $m^{-2}$ cisplatin in patients with advanced NSCLC (Anton et al, 1997). Gemcitabine (1200 mg $m^{-2}$) was administered on days 1, 8 and 15, and cisplatin was given on day 15. All 40 patients were evaluable for response and toxicity. Toxicities were mainly haematological, with grade 3/4 thrombocytopenia seen in 56% of cycles. Partial responses were seen in 19 patients, for an overall response rate of 48%. The median survival was 10.4 months and the 1-year survival rate 35%. Responses and survival rates for all trials are summarized in Table 3.

In order to investigate the effects of escalating the dose of gemcitabine and fractionating the cisplatin administration, a phase I/II study was carried out in 50 patients in Canada by Shepherd et al (1996). Cisplatin was given with each of the weekly doses of gemcitabine. This study established that 1500 mg $m^{-2}$ gemcitabine and 30 mg $m^{-2}$ cisplatin could be given weekly to patients without unacceptable side-effects. Nausea and vomiting were less severe than seen in the other studies, in which higher doses of cisplatin had been administered. Granulocytopenia and thrombocytopenia were, however, frequently observed, and dose reductions were often required. Ten partial responses were seen, giving a response rate of 36%. A phase II study is underway to investigate this regimen further.

In a randomized phase III trial, Cardenal et al (1997) have studied the combination of gemcitabine and cisplatin vs cisplatin and etoposide. A total of 136 patients were enrolled and randomized to receive either gemcitabine (1250 mg $m^{-2}$) on days 1 and 8 or etoposide (100 mg $m^{-2}$) on days 1–3 of a 21-day cycle. Cisplatin (100 mg $m^{-2}$) was given to all patients on day 1 of each cycle. The preliminary response rate for the gemcitabine arm is 48% and the response rate in the etoposide arm is 22%. Toxicities were, in general, less severe in patients receiving the gemcitabine and cisplatin combination, with the exception of grade 3 and 4 thrombocytopenia, which occurred in 20% of patients on this arm, as opposed to 3.5% of patients receiving the etoposide–cisplatin combination.

### COMBINATION OF GEMCITABINE WITH OTHER AGENTS AND WITH RADIOTHERAPY

Ifosfamide is frequently used in the treatment of NSCLC (Schoenike and Dana, 1990). As its mechanism of action and toxicity profile differ from those of gemcitabine, the two drugs were combined in a phase I/II study (Eberhard et al, 1995). In the phase I portion, gemcitabine (1000 mg $m^{-2}$) was given on days 1, 8 and 15 of a 28-day cycle and escalating doses of ifosfamide (1200–1800 mg $m^{-2}$) were given to sequential cohorts on days 8–12. Partial responses were seen, and the dose of ifosfamide identified for phase II investigation was 1500 mg $m^{-2}$. Of the 56 patients enrolled, 50 were evaluable for toxicity and response (Manegold et al, 1996). Grade 3 and 4 neutropenia were seen in 32% and 15% of patients, respectively, and grade 3 and 4 thrombocytopenia in 16% and 3% of patients. Grade 3 alopecia occurred in 57% of patients. Preliminary results suggest that partial responses were seen in 16 patients (32%), suggesting that the combination of gemcitabine and ifosfamide has promising activity in the treatment of NSCLC.

Vinorelbine is another agent active against NSCLC. Krajnik and colleagues (1997) have studied the combination of gemcitabine and vinorelbine in a dose-finding phase I trial in 33 patients with advanced disease. Doses of gemcitabine (1000 mg $m^{-2}$) and vinorelbine (25 mg $m^{-2}$) have not yet given rise to dose-limiting toxicities and investigations are continuing to find the maximum-tolerated dose.

Gemcitabine is a potent radiation sensitizer, with a cytotoxicity enhancement ratio of 1.8 measured in in vitro studies in human colon carcinoma HT-29 cells (Lawrence et al, 1996). The gemcitabine concentrations necessary to induce enhanced sensitivity to radiation were significantly lower than those required to produce direct cytotoxicity (Shewach et al, 1994). This was particularly apparent in pancreas, colorectal, breast and non-small-cell lung cancer cell lines. A phase II trial was designed that combined irradiation (up to 60 Gy) over 6 weeks with weekly injections of gemcitabine (1000 mg $m^{-2}$) in patients with stage III NSCLC (Goor et al, 1996). Eight patients had been recruited when a toxic death resulted in premature termination of the study. Severe side-effects were seen in these patients who had received large doses of both gemcitabine and radiation. Phase I trials are ongoing, using

© Cancer Research Campaign 1998

lower doses of radiation and gemcitabine, to assess the safety and efficacy of this regimen.

The combination of gemcitabine, cisplatin and vinorelbine has been studied in a randomized phase II trial in patients with advanced stage IIIb/IV NSCLC (Comella et al, 1997). On one arm, gemcitabine (1000 mg $m^{-2}$) and cisplatin (50 mg $m^{-2}$) were given on days 1 and 8 every 3 weeks, combined with vinorelbine (25 mg $m^{-2}$). On the second arm, patients were treated with a combination of cisplatin, epirubicin, vindesine and lonidamine. Of the 42 evaluable patients in the gemcitabine arm, one complete response and 25 partial responses were seen, for an overall response rate of 62% (95% CI 46–76%). In the control arm, 37 of the 50 enrolled patients were evaluable for response. Three complete responses and ten partial responses were reported, giving an overall response rate of 35% (95% CI 20–52%). Toxicities were manageable in the gemcitabine arm. This combination is highly effective and will be the subject of a phase III trial.

A phase I/II trial is also underway that will assess the activity of a combination of gemcitabine and carboplatin in NSCLC and, although results are preliminary, this combination appears to be well tolerated (Carmichael et al, 1995).

### CONCLUSION

Gemcitabine, a nucleoside analogue with a novel mechanism of action and single-agent activity against NSCLC, has been used in several different combination regimens in NSCLC. Its use with cisplatin appears to have a significant advantage compared with historical single-agent studies, with response rates of 32–54%, depending on the doses and schedule used. One-year survival rates at 35–61% are particularly encouraging.

The inclusion of ifosfamide in combination therapy has also been associated with an improved response rate (32%) compared with the use of either gemcitabine or ifosfamide as single agents. Trials are also ongoing that will assess the effect of gemcitabine in combination with carboplatin, vinorelbine and radiotherapy.

While the results from all these trials are promising, comparisons need to be made with other combinations currently in use, such as the mitomycin-C–ifosfamide–cisplatin (MIC) regimen (Crinò et al, 1995). Phase III randomized trials are now underway that will compare the gemcitabine–cisplatin (day 2) combination with MIC, as well as with single-agent cisplatin. The results will provide important information on the role of gemcitabine in this disease and will hopefully confirm its value both in terms of improving the activity of existing agents and also in improving survival and quality of life. Its mechanisms of action and low toxicity make it an ideal candidate for consideration in other combinations, and several phase II studies are currently underway. The high activities reported for many of the regimens in advanced disease clearly hold great promise for consideration of their use in the neoadjuvant and adjuvant settings. Major advances in the treatment of NSCLC could be made if the results of surgery are improved or if more patients could be down-staged and become operable; it is to be hoped that the availability of gemcitabine will make an important contribution to advances being made with these approaches.

### REFERENCES

Abratt RP, Bezwoda WR, Goedhals L and Hacking DJ (1997) Weekly gemcitabine with monthly cisplatin: effective chemotherapy for advanced non-small-cell lung cancer. *J Clin Oncol* **15**: 744–749

Anton A, Artal A, Carrato A, Diaz-Fernandez N, González Larriba JL, Vadell C, Masutti B, Montalar J, Aranda E, Barnetto I, Tarazona Y and López-Martín E (1997) Gemcitabine plus cisplatin in advanced NSCLC: final phase II results. *Proc Am Soc Clin Oncol* **16**: 461a

Armand JP, Ducreux M, Mahjoubi M, Abigerges D, Bugat R, Chabot P, Herait P, de Forni M and Rougier P (1995) CPT-II (Irinotecan) in the treatment of colorectal cancer. *Eur J Cancer* **31A**: 1283–1287

Cardenal F, Rosell R, Antón A, Lomas M, Alberola V, Barnetto I, Carrato A, Massuti B, Lianes P, Montalar J, Vadell C, Gonzalez JL and López-Martin E (1997) Gemcitabine + cisplatin in advanced non-small cell lung cancer patients: preliminary randomized phase III results. *Proc Am Soc Clin Oncol* **16**: 458a

Carmichael J, Allerheiligen S and Walling J (1995) A phase I/II study of gemcitabine (GEM) and carboplatin (CBP) in NSCLC. *Proc Am Soc Clin Oncol* **14**: 351

Clarke SJ, Jackman AL and Harrap KR (1991) Antimetabolites in cancer chemotherapy. *Adv Exp Med Biol* **309**: 7–13

Comella P, Panza N, Frasci G, Nicolella GP, Natale M, Pacilio G, Gravina A and Comella G (1997) Gemcitabine (GEM)–cisplatin (CDDP)–vinorelbine (VNR) combination in advanced non-small-cell lung cancer (NSCLC). A phase II randomized study. *Proc Am Soc Clin Oncol* **16**: 449a

Crinò L, Clerici M and Figioli F (1995) Chemotherapy of advanced non-small-cell lung cancer: a comparison of three active regimens. A randomized trial of the Italian Oncology Group for Clinical Research (GOIRC). *Ann Oncol* **6**: 347–353

Crinò L, Scagliotti G, Marangolo M, Figoli F, Clerici M, De Marinis F, Salvati F, Cruciani G, Dogliotti L, Pucci F, Paccagnella A, Adamo V, Altavilla G, Incoronato P, Trippetti M, Mosconi AM, Santucci A, Sorbolini S, Oliva C and Tonato M (1997) Cisplatin–gemcitabine combination in advanced non-small-cell lung cancer: a phase II study. *J Clin Oncol* **15**: 297–303

Eberhard W, Wilke H, Manegold CH, Gatzemeier U, Blatter J, Drings P and Seeber S (1995) Phase I dose finding study of gemcitabine (GEM) and ifosfamide (IFO) in advanced non-small cell lung cancer (NSCLC). *Proc Am Soc Clin Oncol* **14**: 351

Goor C, Scalliet P, Van Meerbeek J, Galdermans D, Groen HJM, Van der Leest AHD, Westerink H and Peolman M (1996) A phase II study combining gemcitabine with radiotherapy in Stage III NSCLC. *Ann Oncol* **7**: 101

Heinemann V, Hertel LW, Grindey GB and Plunkett W (1988) Comparison of the cellular pharmacokinetics and toxicity of 2′,2′-difluorodeoxyclidine and 1-β-D-arabino-furanosylcytosine. *Cancer Res* **48**: 4024–4031

Jolivet J, Cowan KH and Curt GA (1983) The pharmacology and clinical use of methotrexate. *N Engl J Med* **309**: 1094–1104

Kaye SB (1994) Gemcitabine: current status of phase I and II trials. *J Clin Oncol* **12**: 1527–1531

Keating MJ, McCredie KB, Bodey GP, Smith TL, Gehan E and Freircich BJ (1982) Improved prospects for long-term survival in adults with acute myelogenous leukemia. *J Am Med Assoc* **248**: 2481–2486

Krajnik G, Mohn-Staudner A, Marhold F, Malayeri R, Zöchbauer S, Kummer F, Greil R, Huber H and Pirker R for the Austrian Association for the Study of Lung Cancer (1997) Vinorelbine/gemcitabine in advanced non-small cell lung cancer (NSCLC): a phase I trial. *Clin Pharmacol* **16**: 206a

Lawrence TS, Chang EY, Hahnn TM, Hertel LW and Shewach DS (1996) Radiosensitization of pancreatic cancer cells by 2′,2′-difluoro-2′-deoxycytidine. *Int J Radiat Oncol Biol Phys* **34**: 867–872

Manegold CH, Eberhard W, Wilke H, Gatzemeier U, Chomy F, Chomy P, Khayat D, Blatter J and Drings P (1996) Phase II study of gemcitabine (GEM) and ifosfamide (IFO) in advanced non-small cell lung cancer (NSCLC). *Proc Am Soc Clin Oncol* **15**: 380a

Merriman RL, Hertel LW, Schultz RM, Houghton PJ, Rutherford PG, Tanzer LR, Boder GB and Grindey GB (1996) Comparison of the antitumor activity of gemcitabine and ara-C in a panel of human breast, colon, lung and pancreatic xenograft models. *Invest New Drugs* **14**: 243–247

Murphy WK, Fossella FV, Winn RJ, Shin DM, Hynes HE, Gross HM, Davilla E, Leimert J, Dhingra H, Raber MN, Krakoff IH and Hong WK (1993) Phase II study of taxol in patients with untreated non-small-cell lung cancer. *J Natl Cancer Inst* **85**: 388–394

Perigaud C, Gosselin G and Imbach J (1992) Nucleoside analogues as chemotherapeutic agents: a review. *Nucleosides Nucleotides* **11**: 903–945

Peters GJ, Bergman AM, Ruiz van Haperen VWT, Veerman G, Kuiper CM and Braakhuis BJM (1995) Interaction between cisplatin and gemcitabine in vitro and in vivo. *Semin Oncol* **4**: 72–79

Roberts JJ and Pascoe JM (1972) Cross-linking of complementary strands of DNA in mammalian cells by antitumor platinum compounds. *Nature* **235**: 282–284

© Cancer Research Campaign 1998

Rustum YM, Harstrick A, Cao S, Vanhoefer U, Yin MB, Wilke H and Seeber S (1997) Thymidylate synthase inhibitors in cancer therapy: direct and indirect inhibitors. *J Clin Oncol* **15**: 389–400

Sandler AB, Ansari R, McClean J, Fisher W, Dorr A and Einhorn LH (1995) A Hoosier Oncology Group phase II study of gemcitabine plus cisplatin in non-small cell lung cancer. *Proc Am Soc Clin Oncol* **14**:357

Sandler A, Crinò, Steward WP and Abratt RP (1996) Extended survival in stage III and IV non-small cell lung cancer (NSCLC) patients treated with gemcitabine plus monthly cisplatin. *Ann Oncol* **7**: 91

Schoenike SE and Dana WJ (1990) Ifosfamide and mesna. *Clin Pharmacol* **9**: 179–191

Shepherd FA, Burkes R, Cormier Y, Crump M, Feld R, Strack T and Schulz M (1996) Phase I dose-escalation trial of gemcitabine and cisplatin for advanced non-small-cell lung cancer: usefulness of mathematical modelling to determine maximum-tolerable dose. *J Clin Oncol* **14**: 1656–1662

Shewach DS, Hahn TM, Chang E, Hertel LW and Lawrence TS (1994) Metabolism of 2′,2′-difluoro-2′-deoxycytidine and radiation sensitization of human colon carcinoma cells. *Cancer Res* **54**: 3218–3223

Steward WP, Dunlop DJ, Dabouis G, Lacroix H and Talbot D (1996) Phase I/II study of gemcitabine and cisplatin in the treatment of advanced non-small cell lung cancer: preliminary results. *Semin Oncol* **23**: 43–47

© Cancer Research Campaign 1998


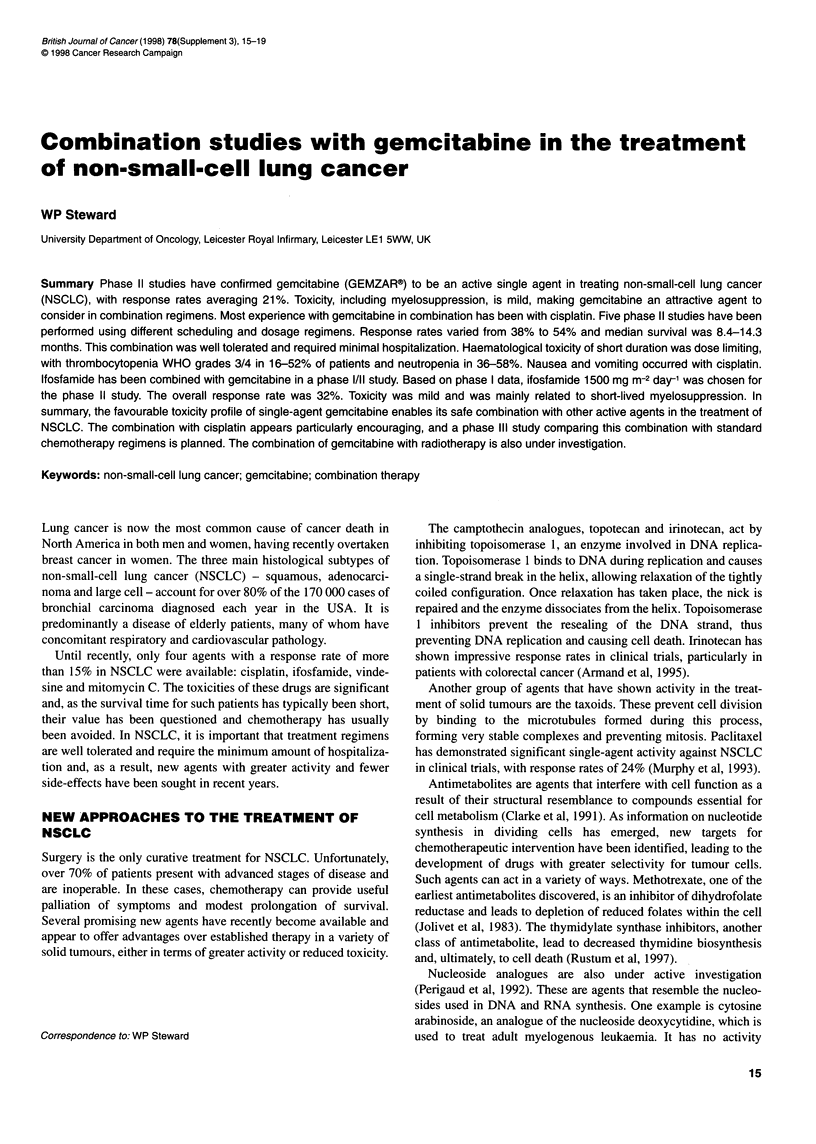

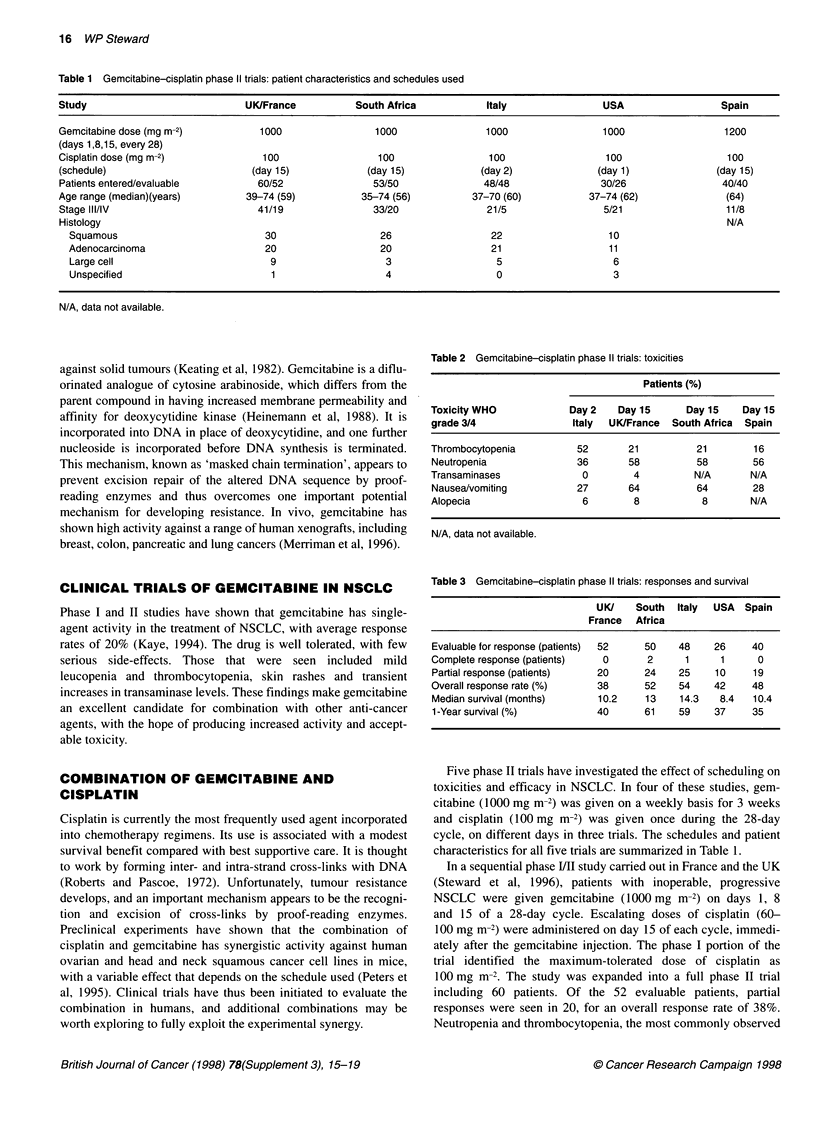

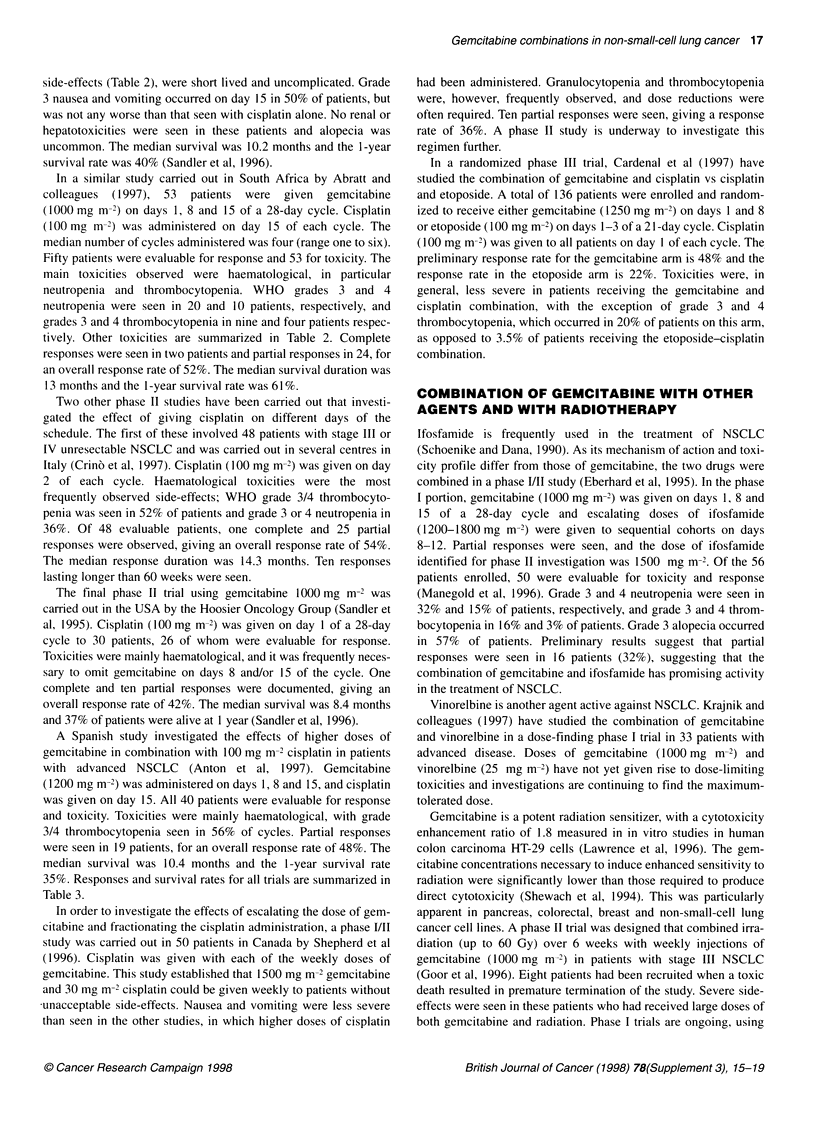

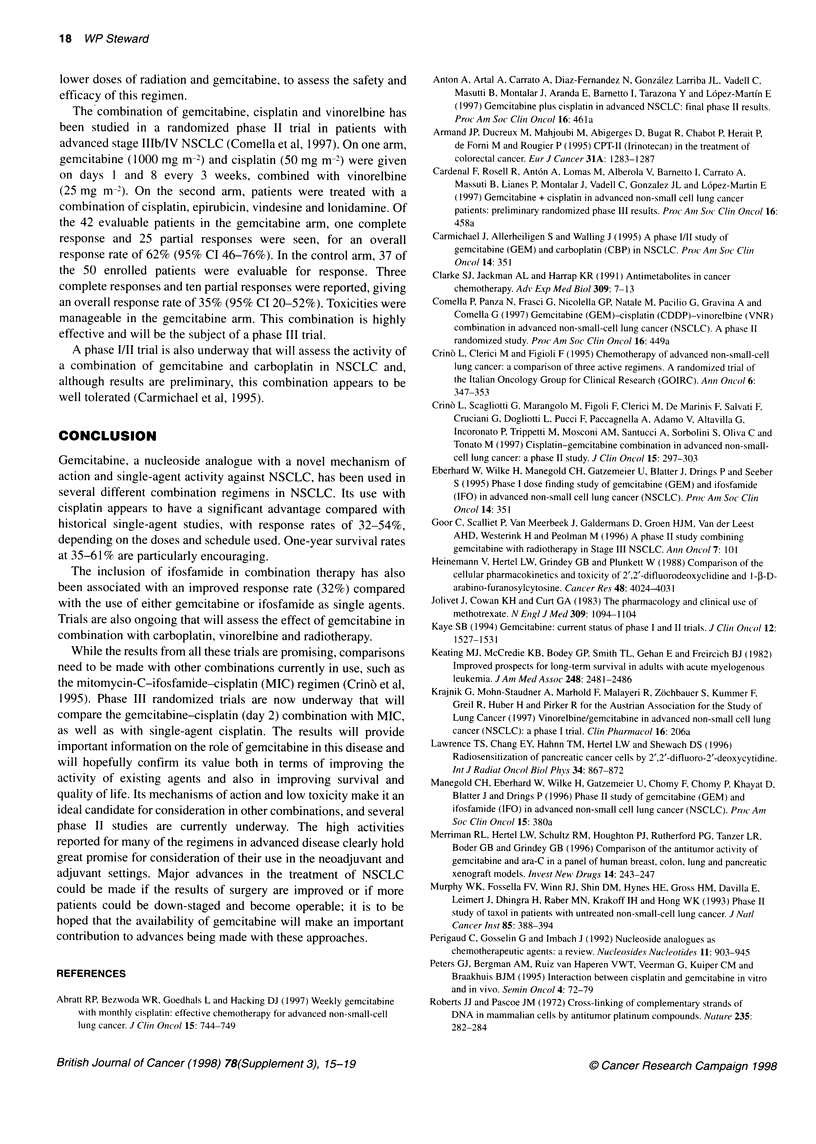

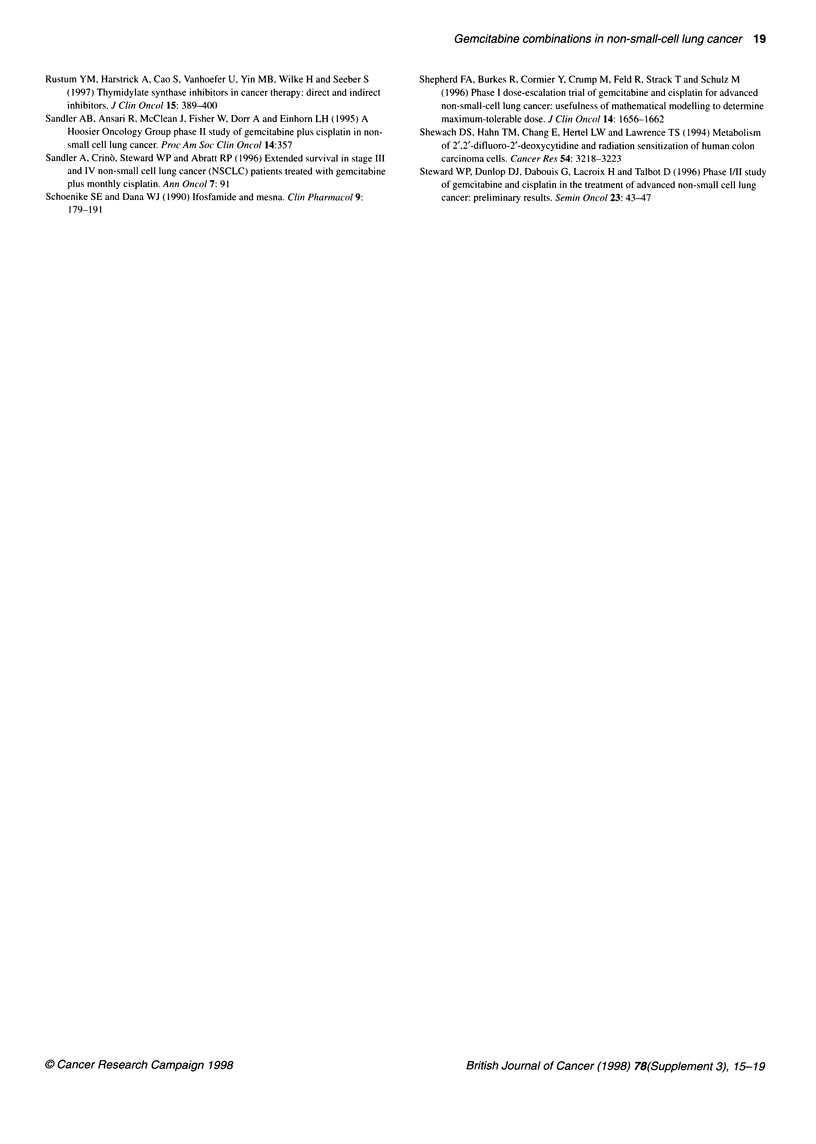


## References

[OCR_00413] Abratt R. P., Bezwoda W. R., Goedhals L., Hacking D. J. (1997). Weekly gemcitabine with monthly cisplatin: effective chemotherapy for advanced non-small-cell lung cancer.. J Clin Oncol.

[OCR_00424] Armand J. P., Ducreux M., Mahjoubi M., Abigerges D., Bugat R., Chabot G., Herait P., de Forni M., Rougier P. (1995). CPT-11 (irinotecan) in the treatment of colorectal cancer.. Eur J Cancer.

[OCR_00442] Clarke S. J., Jackman A. L., Harrap K. R. (1991). Antimetabolites in cancer chemotherapy.. Adv Exp Med Biol.

[OCR_00452] Crinò L., Clerici M., Figoli F., Carlini P., Ceci G., Cortesi E., Carpi A., Santini A., Di Costanzo F., Boni C. (1995). Chemotherapy of advanced non-small-cell lung cancer: a comparison of three active regimens. A randomized trial of the Italian Oncology Group for Clinical Research (G.O.I.R.C.).. Ann Oncol.

[OCR_00462] Crinò L., Scagliotti G., Marangolo M., Figoli F., Clerici M., De Marinis F., Salvati F., Cruciani G., Dogliotti L., Pucci F. (1997). Cisplatin-gemcitabine combination in advanced non-small-cell lung cancer: a phase II study.. J Clin Oncol.

[OCR_00477] Heinemann V., Hertel L. W., Grindey G. B., Plunkett W. (1988). Comparison of the cellular pharmacokinetics and toxicity of 2',2'-difluorodeoxycytidine and 1-beta-D-arabinofuranosylcytosine.. Cancer Res.

[OCR_00482] Jolivet J., Cowan K. H., Curt G. A., Clendeninn N. J., Chabner B. A. (1983). The pharmacology and clinical use of methotrexate.. N Engl J Med.

[OCR_00486] Kaye S. B. (1994). Gemcitabine: current status of phase I and II trials.. J Clin Oncol.

[OCR_00490] Keating M. J., McCredie K. B., Bodey G. P., Smith T. L., Gehan E., Freireich E. J. (1982). Improved prospects for long-term survival in adults with acute myelogenous leukemia.. JAMA.

[OCR_00502] Lawrence T. S., Chang E. Y., Hahn T. M., Hertel L. W., Shewach D. S. (1996). Radiosensitization of pancreatic cancer cells by 2',2'-difluoro-2'-deoxycytidine.. Int J Radiat Oncol Biol Phys.

[OCR_00521] Murphy W. K., Fossella F. V., Winn R. J., Shin D. M., Hynes H. E., Gross H. M., Davilla E., Leimert J., Dhingra H., Raber M. N. (1993). Phase II study of taxol in patients with untreated advanced non-small-cell lung cancer.. J Natl Cancer Inst.

[OCR_00530] Peters G. J., Bergman A. M., Ruiz van Haperen V. W., Veerman G., Kuiper C. M., Braakhuis B. J. (1995). Interaction between cisplatin and gemcitabine in vitro and in vivo.. Semin Oncol.

[OCR_00535] Roberts J. J., Pascoe J. M. (1972). Cross-linking of complementary strands of DNA in mammalian cells by antitumour platinum compounds.. Nature.

[OCR_00544] Rustum Y. M., Harstrick A., Cao S., Vanhoefer U., Yin M. B., Wilke H., Seeber S. (1997). Thymidylate synthase inhibitors in cancer therapy: direct and indirect inhibitors.. J Clin Oncol.

[OCR_00559] Schoenike S. E., Dana W. J. (1990). Ifosfamide and mesna.. Clin Pharm.

[OCR_00563] Shepherd F. A., Burkes R., Cormier Y., Crump M., Feld R., Strack T., Schulz M. (1996). Phase I dose-escalation trial of gemcitabine and cisplatin for advanced non-small-cell lung cancer: usefulness of mathematic modeling to determine maximum-tolerable dose.. J Clin Oncol.

[OCR_00570] Shewach D. S., Hahn T. M., Chang E., Hertel L. W., Lawrence T. S. (1994). Metabolism of 2',2'-difluoro-2'-deoxycytidine and radiation sensitization of human colon carcinoma cells.. Cancer Res.

[OCR_00575] Steward W. P., Dunlop D. J., Dabouis G., Lacroix H., Talbot D. (1996). Phase I/II study of gemcitabine and cisplatin in the treatment of advanced non-small cell lung cancer: preliminary results.. Semin Oncol.

